# Core-shell of FePt@SiO_2_-Au magnetic nanoparticles for rapid SERS detection

**DOI:** 10.1186/s11671-015-1111-0

**Published:** 2015-10-22

**Authors:** Andri Hardiansyah, An-Yu Chen, Hung-Liang Liao, Ming-Chien Yang, Ting-Yu Liu, Tzu-Yi Chan, Hui-Ming Tsou, Chih-Yu Kuo, Juen-Kai Wang, Yuh-Lin Wang

**Affiliations:** Department of Materials Science and Engineering, National Taiwan University of Science and Technology, Taipei, 10607 Taiwan; Department of Materials Engineering, Ming Chi University of Technology, New Taipei City, 24301 Taiwan; Institute of Polymer Science and Engineering, National Taiwan University, Taipei, 10617 Taiwan; Center for Condensed Matter Sciences, National Taiwan University, Taipei, 10617 Taiwan; Institute of Atomic and Molecular Sciences, Academia Sinica, Taipei, 10617 Taiwan; Department of Physics, National Taiwan University, Taipei, 10617 Taiwan

**Keywords:** Core-shell nanoparticles, Surface-enhanced Raman scattering, Magnetic separation, Bio-detection

## Abstract

In this study, multifunctional hybrid nanoparticles composed of iron platinum (FePt), silica (SiO_2_), and gold nanoparticles (AuNPs) had been developed for surface-enhanced Raman scattering (SERS) application. Core-shell structure of SiO_2_ and FePt nanoparticles (FePt@SiO_2_) was fabricated through sol-gel process and then immobilized gold nanoparticles onto the surface of FePt@SiO_2_, which displays huge Raman enhancement effect and magnetic separation capability. The resulting core-shell nanoparticles were subject to evaluation by transmission electron microscopy (TEM), Energy-dispersive X-ray spectroscopy (EDX), zeta potential measurement, and X-ray photoelectron spectroscopy (XPS). TEM observation revealed that the particle size of resultant nanoparticles displayed spherical structure with the size ~30 nm and further proved the successful immobilization of Au onto the surface of FePt@SiO_2_. Zeta potential measurement exhibited the successful reaction between FePt@SiO_2_ and AuNPs. The rapid SERS detection and identification of small biomolecules (adenine) and microorganisms (gram-positive bacteria, *Staphylococcus aureus*) was conducted through Raman spectroscopy. In summary, the novel core-shell magnetic nanoparticles could be anticipated to apply in the rapid magnetic separation under the external magnetic field due to the core of the FePt superparamagnetic nanoparticles and label-free SERS bio-sensing of biomolecules and bacteria.

**Electronic supplementary material:**

The online version of this article (doi:10.1186/s11671-015-1111-0) contains supplementary material, which is available to authorized users.

## Background

Rapid, specific, accurate, and sensitive identification and detection of small molecules and pathogenic bacteria are essential toward clinical treatment of infectious diseases. Traditional approaches for the pathogen bacteria identification often requires time-consuming bacteria culture and amplification protocols. Conventional protocols for microbial detection, though reliable and gold standard, are time and cost consuming and inconvenient for field situation [1, 2]. In this respect, sensitivity of bacteria into antibiotic depends mostly on measuring the change of its proliferation in response to the drug. Thus, in order to overcome these challenges, there is a development of new protocols and its combination with nanotechnology as a new method for the higher sensitivity and low-cost strategies for fast bio-detection and identification of small molecules or bacteria [1, 3, 4].

Raman spectroscopy elaborates the molecular vibrational signal to be used to identify and detect the molecular species such as small molecules and bacteria. Surface-enhanced Raman scattering (SERS) technique has attracted a lot of attention for more than three decades [5–7] because it enhances the Raman signal of the small molecules and or bacteria by several orders of magnitude. SERS nanotechnology could observe the monolayer (single-molecule level) [8] and studies on the identification of biological species, i.e., three kinds species of microbes (gram-positive, gram-negative, and mycobacteria), which have been reported in our previous work [3, 5]. The enhancement is known to develop from the strong optical intensity localized between the surface of metallic nanostructure [8]. SERS takes advantage of localized surface plasmon resonance (LSPR) in nanoscale systems based on metallic nanoparticles such as silver and gold. The enhancements ranging from a factor of 10^6^ to 10^16^ have been reported in various studies [9–11]. The mechanisms for this enhancement in SERS are attributed to electromagnetic field enhancement due to localized surface plasmon resonance and also to chemical interactions of the analytic molecules with the substrate moiety. As a result of these enhancements, SERS can have the sensitivity for the identification of ultra-trace levels of analytes to single molecules and bacteria. This level of sensitivity is useful for the detection of a small number of analytic molecules normally found in a cellular compartment [12]. Recently, the development of SERS active particles have been successfully used as labels or probes in immunoassays, bacteria, and DNA detection [3, 13–18].

The incorporation of nanoparticles through self-assembly mechanism provides an interesting characteristic in nanotechnology application. Core-shell nanoparticles have emerged as an important type of functional nanostructures with promising applications in many fields, especially in health sciences and biomaterials applications. Nanoparticles have also been assembled and bound to functionalized moiety with various mechanisms [19].

Various nanostructures have been synthesized in diverse formulations and functionalization whether using a single or multiple nanoparticles. For instance, iron oxide nanoparticles have been used for cellular separation and contrast agent. Silver nanoparticles have been used for bactericidal and SERS detection agent. Gold nanoparticles have been used for imaging, photothermal therapy, and LSPR detection. The most common and well investigated metallic nanoparticles, gold nanoparticles hold particularly interesting characteristics. Gold nanoparticles were applied in the significant place in medicine, material sciences, as well as diagnostics for their unique optical and physiochemical properties [1, 20–25]. Their facile synthesis and bio-conjugation procedures, along with its unique surface plasmon properties, made gold nanoparticles practicable in labs without expensive or sophisticated equipment [26–28].

Moreover, gold nanoparticles have also been combined with various nanostructures in order to develop SERS substrate [27, 29]. In this work, the core-shell nanoparticles were developed by functionalized amino-silane around FePt nanoparticles (FePt@SiO_2_) nanoparticles and then grated the gold nanoparticles onto the surfaces of FePt@SiO_2_ nanoparticles. Systematic characterizations have been conducted in order to elaborate the morphology and chemical properties of the hybrid nanoparticles. The coverage of the gold nanoparticles and clusters on the surfaces of the silica nanoparticles was evaluated using transmission electron microscopy (TEM). Zeta potential measurement was conducted to evaluate the surface charges of nanoparticles. Furthermore, SERS detection was conducted toward adenine and *Staphylococcus aureus* to elaborate the Raman enhancement signals using the novel core-shell nanoparticles.

## Methods

### Materials

Iron (II) chloride tetrahydrate (FeCl_2_.4H_2_O) (>99 %), platinum (II) acetylacetonate, (Pt(acac)_2_) (>99.9 %), oleylamine (*cis*-1-amino-9-octadecene (≥98 %), oleic acid (*cis*-9-octadecenoic acid (>99 %), benzyl ether, cyclohexane (99.5 %)), IGEPAL® CA-520 (octylphenoxy poly(ethyleneoxy) ethanol, branched), tetraethyl orthosilicate (TEOS) (99.99 %), ammonia (≥99 %), *N*-[3-(trimethoxysilyl) propyl] ethylenediamine (energy-dispersive X-ray spectroscopy (EDS)) (97 %), acetic acid (CH_3_COOH, ≥99 %), trisodium citrate dihydrate (Na_3_C_6_H_5_O_7_ · 2H_2_O, ≥99.5 %), hydrogen tetrachloroaurate (III) trihydrate (HAuCl_4_ · 3H_2_O, 99 %), and adenine (C_5_H_5_N_5_, ≥99 %) were purchased from Sigma Aldrich, St. Louis, MO, USA. Ethyl alcohol (ethanol, analytical standard) was purchased from Fluka Analytical, USA. Agar Bacteriological was purchased from Oxoid. Luria-Bertani (LB Broth) was purchased from Difco^TM^. High-purity water purified by a Milli Q Plus water purifier system (Milipore, USA), with a resistivity of 18.3 M Ω cm, was used in all experiments. All the chemicals were used without further purification.

### Synthesis of Iron Platinum Nanoparticles

**Fig. 1:**
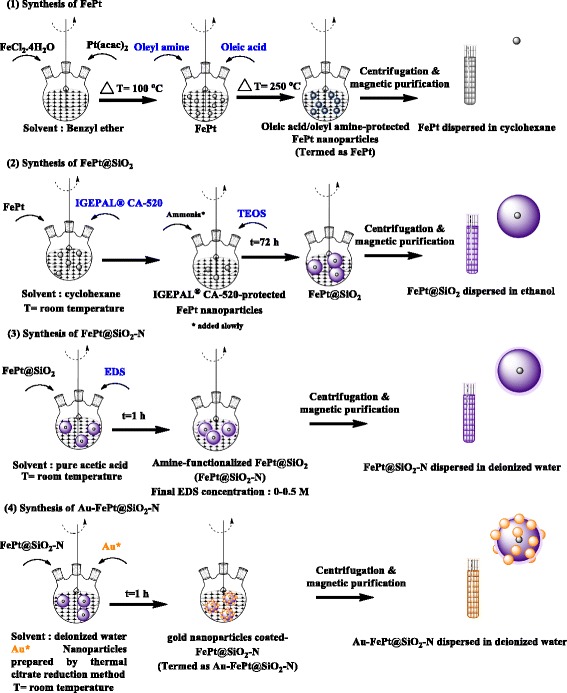
Schematic diagrams of the fabrication of FePt, FePt@SiO_2_, FePt@SiO_2_-N, and Au-FePt@SiO_2_-N nanoparticles

Iron platinum (FePt) nanoparticles were synthesized through standard airless procedures. Briefly, FeCl_2_.4H_2_O (396 mg) and platinum (II) acetylacetonate (Pt(acac)_2_) (196 mg) were mixed in benzyl ether (30 mL) with vigorous stirring under nitrogen atmosphere for 20 min. Further, the solution was heated to 100 °C. After reaching 100 °C, oleic acid (170 μL) and oleylamine (160 μL) were mixed into the solution under vigorous stirring for 20 min. Further, the solution was heated to 250 °C for 30 min which resulted in black colored solution of FePt. The solution was allowed to cool to room temperature. Purification was carried out by washing several times with ethanol (FePt: ethanol = 1:4 *v/v*) then centrifuged 6000 rpm for 20 min. The as-synthesized FePt nanoparticles were separated magnetically using a strong neodymium magnet. Dark yellow supernatant was discarded. The precipitate was uniformly dispersed in cyclohexane (Fig. 1).

### Preparation of FePt@SiO_2_

FePt nanoparticles were encapsulated into the silica nanoparticles through sol-gel processes. Briefly, 10 mL of FePt nanoparticles (26.5 mg/mL) and 8 ml of IGEPAL® CA-520 were mixed with 170 mL cyclohexane under vigorous stirring. Afterwards, 1.3 mL ammonia (30 %) was added slowly to the mixture followed by adding 1 mL triethoxysilane (TEOS), and the reaction was mixed under vigorously stirring for 72 h. After 72 h, the encapsulated particles were mixed with ethanol and centrifuged 4500 rpm for 30 min to remove the excess silica formed during the hydrolysis-condensation processes. The resultant of nanoparticles was separated using a strong neodymium magnet and then dissolve in ethanol. The product termed as FePt@SiO_2_.

### Surface Modification of FePt@SiO_2_ Using EDS Reaction

The *N*-[3-(trimethoxysilyl) propyl] ethylenediamine (EDS) was used for surface modification of FePt@SiO_2_. Briefly, an equal volume of FePt@SiO_2_ (183 mg/mL) and acetic acid were mixed with EDS under vigorously stirring for 1 h at room temperature which will resulted in amine-functionalized FePt@SiO_2_ (FePt@SiO_2_-N). The solution was subject to centrifugation at 4500 rpm for 30 min to remove any traces. The resulting nanoparticles were collected using a strong neodymium magnet and then dissolve in deionized water. FePt@SiO_2_-N with different concentration of EDS in the range of 0–0.5 M was prepared.

### Preparation of Gold Nanoparticles

Gold nanoparticles were prepared through well-established citrate reduction techniques as method described previously with minor modification [30]. In a typical experiment, 103 mL of HAuCl_4_ 0.33 mM was place on a three-neck round-bottom flask on a stirring hot plate. To the rapidly stirred boiling solution, 3.5 mL of a 1 % solution of trisodium citrate dihydrate (Na_3_C_6_H_5_O_7_.2H_2_O) was quickly added. The gold sol gradually forms as the citrate reduces the gold (III). It was remove from heat when the solution has turned deep red. The product was termed as gold nanoparticles (AuNPs).

### Preparation of Au-FePt@SiO_2_-N

Gold-surface modified FePt@SiO_2_ was prepared through the simple mixing of gold solution and FePt@SiO_2_-N. Briefly, FePt@SiO_2_-N and Au NPs were mixed under vigorously stirring for 1 h at room temperature. The resulting nanoparticles were then centrifuged at 4500 rpm for 30 min to remove any traces and impurities. The resultant termed as gold nanoparticles-modified FePt@SiO_2_-N (Au-FePt@SiO_2_-N). Au-FePt@SiO_2_-N with different Au concentration in the range of 0–238 μM was prepared.

### Characterizations

Structure and morphology of the resultant nanoparticles were characterized by transmission electron microscopy using TEM-8100, Hitachi, at an acceleration voltage of 200 kV. Prior to the TEM observation, an aliquot of suspension of samples was diluted with water until optically clear. Briefly, an aliquot of suspension were placed on a carbon-coated Formvar copper grid (300 mesh, Electron Microscopy Sciences) to form a thin film specimen. The excess of the sample was blotted using a filter paper then air-dried overnight in a dust-free area before loading into the specimen chamber. Elemental analysis was performed using EDS analysis system attached to the same instrument. Zeta (ζ) potential was determined through electrophoretic mobility measurement and calculated using Helmholtz-Smoluchowski’s equation. The zeta (ζ) potential of the samples were determined at 25 °C by using dynamic light scattering (DLS) spectrophotometer, Horiba Instrument, Horiba, Kyoto, Japan. All characterization measurements were repeated three times. The resultant of nanoparticles were further investigated using X-ray photoelectron spectroscopy (XPS) (ESCALAB 250, Thermo VG Scientific, West Sussex, UK) equipped with Mg K_α_ at 1253.6 eV at the anode to evaluate their chemical binding energy characteristics. Briefly, an aliquot of solution was put on silicon slice and dried overnight. Furthermore, the sample was then placed to XPS chamber and excited with X-rays with a monochromatic Al K_α1,2_ radiation. Magnetization as a function of the field was evaluated using a vibrating sample magnetometer (VSM) Lakeshore model 7400 at room temperature.

### Bacteria Culture

*S. aureus* (ATCC 6538P) was used as the model of pathogenic bacteria. Briefly, frozen preserved stock was thawed at room temperature, and then 0.1 mL were pipetted and streaked into a quadrant on a nutrient agar plate. Nutrient agar was composed of 6 g Agar Bacteriological (Agar No. 1) LP0011 powder mixed with 10 g Difco™ LB Broth, Miller (Luria-Bertani) powder and diluted with 400 mL deionized (DI) water and cultured at 37 °C for 18–24 h to allow the formation of colonies. Afterward, a single colony was scraped with a loop and swabbed onto a 15°-slant medium (nutrient agar) and then incubated at 37 °C for 18–24 h.

### Surface-enhanced Raman Spectroscopy

Raman spectroscopy was conducted using Raman microscope instrument (HORIBA Jobin Yvon S.A.S.) equipped with laser (*λ* = 633 nm). The instrument was calibrated against a Si crystal. Prior to the experiment, an aliquot of Au-FePt@SiO_2_-N solution was mixed equally with the concentrations of adenine (5 × 10^−5^ M) or *S. aureus* (5 × 10^6^ CFU/mL) and then homogenized using vortex (VTX-3000 L Mixer Uzusio, LMS Co., Tokyo, Japan). Afterwards, the mixed suspension was dropped wisely into the alumina chip followed by drying in free dust-air atmosphere.

## Results and Discussion

### Structure and Morphology of FePt Nanoparticles

**Fig. 2:**
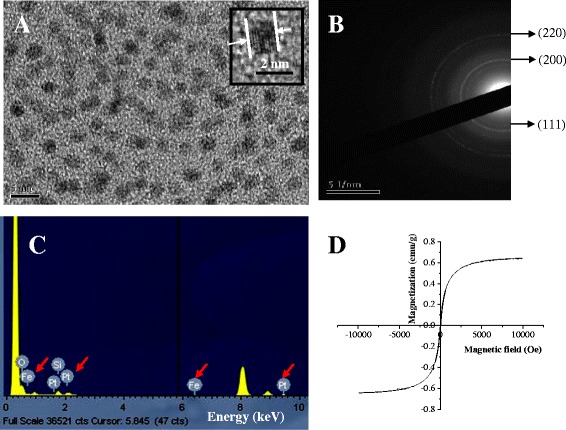
**a** TEM image, **b** selective area diffraction (SAD) pattern, **c** EDX spectrum (the red arrow symbols show that Fe and Pt atoms in the nanoparticles), and **d** magnetic hysteresis curve of as-syntesized FePt nanoparticles

TEM images display homogeneously distribution of FePt nanoparticles, which are surface-protected by oleic acid and oleyl amine, and acted as ligands to stabilize FePt nanoparticles, as shown in Fig. 2a. Furthermore, oleic acid and oleic amine protecting the surface of FePt nanoparticles would further generate the hydrophobic characteristic of FePt nanoparticles [31]. The as-synthesized FePt nanoparticles could disperse homogeneously in the nonpolar solvents such as cyclohexane, due to the presence of oleic acid and oleylamine. The average diameter of the FePt nanoparticles was determined to be 2–3 nm. The insert of TEM image revealed the crystalline nature of FePt nanoparticles. The lattice fringes of the core are clearly shown in the image, corresponding to (111) lattice planes for FePt nanoparticles (Fig. 2b) [32]. Energy-dispersive X-ray spectroscopy (EDX) analysis shows that the particles contain the elements of Fe and Pt (Fig. 2c). VSM measurement revealed that FePt nanoparticles exhibited superparamagnetic characteristics with a single magnetic domain and exhibit magnetic moment in the presence of external magnetic field. When magnetic field is removed, the particles return to their nonmagnetic nature immediately (Fig. 2d).

### Physicochemical Properties of FePt@SiO_2_, FePt@SiO_2_-N, and Gold Nanoparticles

**Fig. 3:**
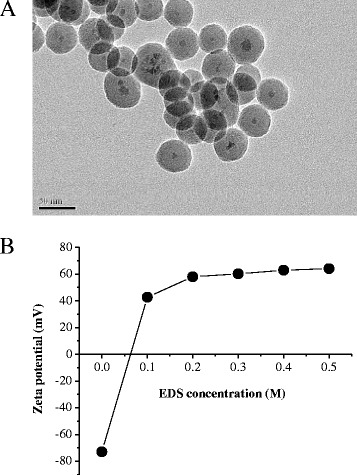
**a** TEM images of FePt@SiO_2_. **b** Zeta potential of FePt@SiO_2_-N with various EDS concentrations

Figure 3a shows the core-shell TEM images of FePt@SiO_2_. It revealed the successful sol-gel reaction of the shell of silica (SiO_2_) (25–30 nm) for encapsulation of the core of FePt nanoparticles. Most of the SiO_2_ spheres encapsulated one FePt nanoparticle, and a small portion of the particles contains either zero or two FePt nanoparticles. The different contrast between the core and shell region is due to the different electron penetration on metallic FePt nanoparticles and SiO_2_ nanoparticle [32]. Moreover, FePt@SiO_2_ nanoparticles could easily dispersed in water system. Surface modification of FePt@SiO_2_ was developed through the reaction with *N*-[3-(trimethoxysilyl) propyl] ethylenediamine (EDS), called as FePt@SiO_2_-N. The structure and morphology of FePt@SiO_2_-N were also developed in the spherical structure (Additional file 1: Figure S1A). Figure 3b shows the zeta potential of amine-functionalized FePt@SiO_2_ (FePt@SiO_2_-N). Zeta potential measurement revealed that the surface charge of the FePt@SiO_2_ nanoparticles, which would increase with the addition of EDS concentration increase, due to the positive charge (amine group) of EDS. After surface modification, the FePt@SiO_2_-N nanoparticles are overall positively charged with the average zeta potential range from +40 until +60 mV Furthermore, we have successfully fabricate AuNPs through well-established citrate reduction method, and TEM images of gold nanoparticles revealed the spherical morphology in the diameter of ~17 nm (Additional file 1: Figure S1B).

### Structure and Morphology of Au-FePt@SiO_2_-N

**Fig. 4:**
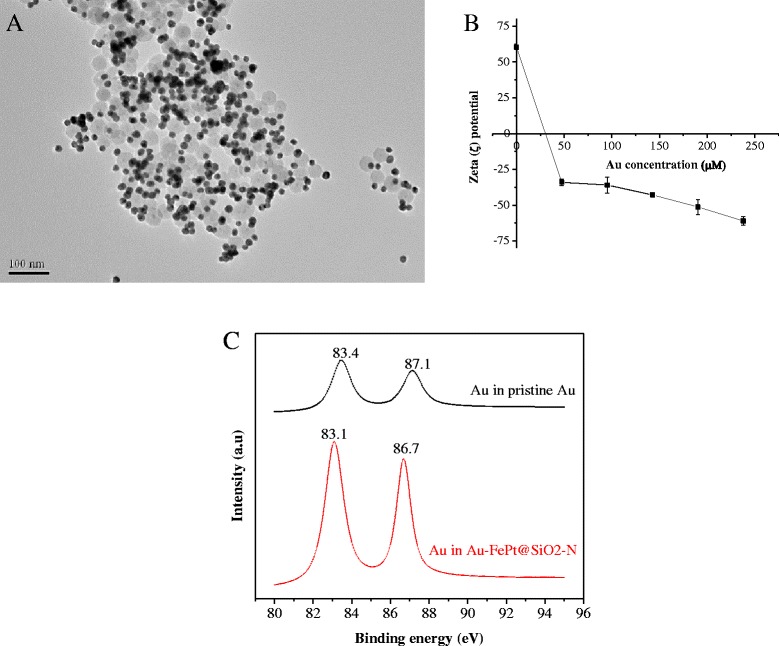
**a** TEM images of Au-FePt@SiO_2_-N (0.3 M of EDS and 142.8 μM of Au nanoparticles), **b** Zeta potential of FePt@SiO_2_-N with various gold nanoparticles concentrations, and **c** XPS analysis (Au-4f) of pristine gold nanoparticles and Au-FePt@SiO_2_-N

FePt@SiO_2_-N could electrostatically attract negatively charged of Au nanoparticles (~17 nm) synthesized in advance by citrate reduction method of HAuCl_4_ with trisodium citrate dihydrate (Na_3_C_6_H_5_O_7_.2H_2_O). This result is in accordance with the previously reported research [19, 33]. As shown in Fig. 4a, TEM images of extensively washed Au-FePt@SiO_2_-N nanoparticles revealed a dense surface coverage of gold nanoparticles. The attractive force between Au nanoparticles and FePt@SiO_2_-N is sufficiently strong, which could tightly and stably attach each other, no matter how the FePt@SiO_2_-N nanoparticles are centrifuged and re-dispersed by ultrasonication. Lower concentrations of EDS and gold nanoparticles exhibited a single nanoparticle attached on the FePt@SiO_2_-N, where individual gold nanoparticles are homogenously separated, as shown in SEM image (Additional file 1: Figures S2B, D and S3B, D). In contrast, higher concentration of EDS and gold nanoparticles show the clusters structure (Additional file 1: Figures S2E, F and S3E, F). The optimum concentration of Au-FePt@SiO_2_-N was found in 0.3 M of EDS and 142.8 μM of gold nanoparticles, which displays homogenously distribution of Au nanoparticles.

Figure 4b shows the zeta potentials of Au-FePt@SiO_2_-N with various gold concentrations. It revealed that the surface charged of the core-shell nanoparticles becomes negatively charged after gold immobilization by zeta potential measurement. This might be that the Au-trisodium citrate dihydrate nanoparticles coverage of FePt@SiO_2_-N develops the net negatively charged nanoparticles, which further prove the successful immobilization of Au nanoparticles onto the surface of FePt@SiO_2_-N. Figure 4c displays the region of the XPS spectra corresponding to Au-4f. Pristine gold nanoparticles exhibited binding energy of Au 4f_7/2_ and Au 4f_5/2_ at 83.4 and 87.1 eV, respectively. These peaks were shifted to 83.1 (Au-4f_7/2_) and 86.7 eV (Au-4f_5/2_) in the Au-FePt@SiO_2_-N. The shifted peaks further confirmed the reaction between gold (Au) nanoparticles and FePt@SiO_2_-N nanoparticles through electrostatic interaction.

### SERS Detection of Adenine

**Fig. 5:**
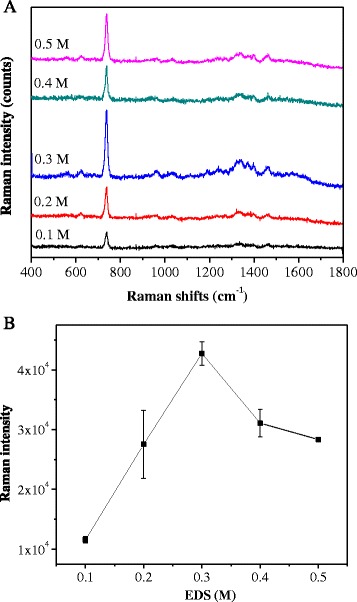
(**a**) SERS spectra of adenine; (**b**) integrated intensity (733 cm^−1^) of SERS spectra in the Au-FePt@SiO_2_-N with various EDS concentrations (0.1–0.5 M)

The advantage to use the nanoparticle is its high-surface area could increase the interaction between adenine and the Au-FePt@SiO_2_-N for surface-enhanced Raman scattering (SERS) bio-detection. Figure 5 shows the Raman spectrum of adenine in the various concentrations of Au-FePt@SiO_2_-N with EDS concentration ranging from 0.1 to 0.5 M. Increasing EDS concentration of Au-FePt@SiO_2_-N from 0 to 0.3 M will increase the Raman intensity (integrated peak at ~733 cm^−1^) of adenine. The integrated peak at ~733 cm^−1^ is assigned to the ring-breathing vibration of adenine [34]. However, Raman intensity slightly decreased at 0.4 and 0.5 M of EDS concentration. This reason could be explained that EDS play an important role in the surface modification of FePt@SiO_2_, which is further developed as a site for the reaction between the FePt@SiO_2_ and gold nanoparticles (Au). This result is in agreement with the TEM results. At 0.3 M of EDS concentration, the gold nanoparticles could homogeneously disperse on the surface of FePt@SiO_2_-N, thus, developed an optimum “hot spot” effect for Raman enhancement signals of adenine. High concentration of EDS could develop the high entrapment of gold nanoparticles become a gold clustering system. However, as the increasing of nanoparticles size and concentration, the convex shape of the surface becomes flatter, the particles could absorb less light and less inelastic scattering pronounce on the surface, which tend to weakening of electromagnetic field on the surface and the overall SERS intensity [35].

**Fig. 6:**
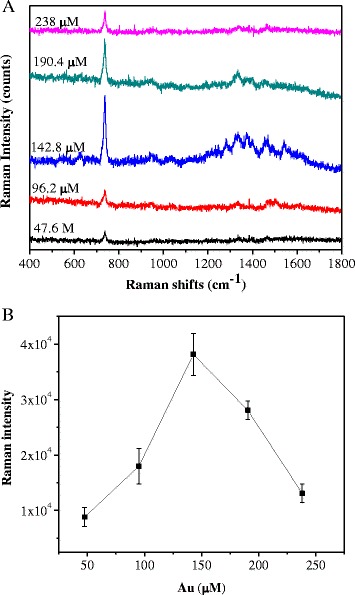
(**a**) SERS spectra of adenine; (**b**) integrated intensity (733 cm^−1^) of SERS spectra in the Au-FePt@SiO_2_-N (0.3 M of EDS) with various Au nanoparticles concentrations (47.6–238 μM)

Figure 6 shows the Raman spectrum of adenine in various concentration of Au-FePt@SiO_2_-N ranging from 47.6 to 238 μM. The optimum Raman enhancement signal was found in the 142.8 μM of Au-FePt@SiO_2_-N. Raman signal of adenine slightly reduced at higher concentration of Au-FePt@SiO_2_-N (190.4–238 μM). The result and mechanism are similar with the abovementioned EDS concentrations. The enhancement effect (EF) at 733 cm^−1^ of “adenine” on the Au-FePt@SiO2-N SERS substrate are determined using the following expression: EF = (*I*_SERS_/*N*_SERS_)/(*I*_bulk_/*N*_bulk_), where *I*_SERS_ is the intensity of a vibrational mode in the surface-enhanced spectrum, *I*_bulk_ is the intensity of the same mode in the Raman spectrum, *N*_SERS_ is the number of molecules adsorbed on the SERS substrate, and *N*_bulk_ is the number of molecules in the bulk. *N*_SERS_ can be obtained according to the method proposed by Orendorff et al. [36]. The enhancement factor of Au-FePt@SiO_2_-N (0.3 M of EDS and 142.8 μM of gold nanoparticles) is about 2 × 10^7^ magnification.

### SERS Detection of Bacteria (*S. aureus*)

**Fig. 7:**
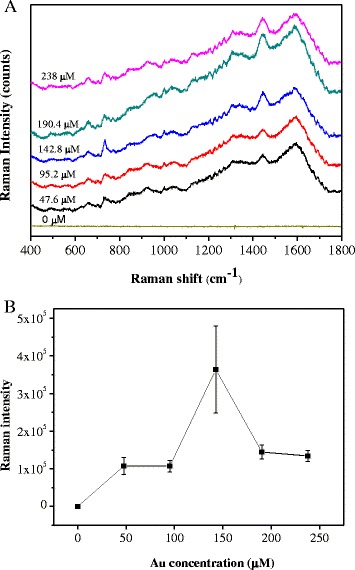
(**a**) SERS spectra of *S. aureus*; (**b**) integrated intensity (735 cm^−1^) of SERS spectra in the Au-FePt@SiO_2_-N (0.3 M of EDS) with various Au nanoparticles concentrations (47.6–238 μM)

There is currently increasing significant development of SERS nanotechnology for the rapid characterization and identification of bacteria. SERS nanotechnology could observe the chemical molecular components of the bacteria cell walls and provide the chemical identification of bacteria for clinical diagnostics. In this respect, the surface characteristics and the affinity between samples and SERS nanohybrids are important, which would affect the detected limitation of SERS signals and the intensities of the SERS spectra. Thus, if the bacterial cell walls can effectively been attached to the surface of SERS nanohybrids, it will contribute to highly enhanced Raman signal [4, 23]. Vibrational bands of nucleic acids, proteins, phospholipids, and polysaccharides are anticipated to contribute to the Raman spectra. For example, the strong bacterial SERS band at ~735 cm^−1^ has been attributed to the nucleic acid base adenosine [24, 37]. The SERS spectra for *S. aureus* (0.5 × 10^7^ CFU/mL) assays observed by using Au-FePt@SiO_2_-N with various gold nanoparticles concentration are shown in Fig. 7, which displays a typical SERS response after the incubation with *S. aureus*. The spectra contain features which are attributable to SERS of *S. aureus* and are dominated by bands that are representative of the bacteria membrane protein. The outer bacteria cell layer sensitivity of this spectroscopic method makes the assignment of this band and features at 965, 1030, and 1080 cm^−1^ more likely developed from the lipid layer components of the cell walls and membranes. Raman signal at 715 cm^−1^ (C-N stretching) and 1060 cm^−1^ (C-C stretching) are well-known markers of lipid layer fluidity, and vibrational bands in the 930–1130 cm^−1^ region have been also previously assigned to membrane phospholipids. Amide I, II, and III vibrations, associated with protein backbone, and carboxylic stretches, are expected in the 1310–1589 cm^−1^ region and probably dominate the SERS vibrational signature in this region [24]. As shown in Fig. 7, the optimum enhancement of SERS signals could be reached using 142.8 μM gold nanoparticles in Au-FePt@SiO_2_-N. This might be due to the increasing gold concentration that could increase the surface area of the nanoparticles then provide the larger sites for bacteria entrapment thus increasing the Raman signal of *S. aureus*, and also it might be due to the development an appropriate hot spot effect for enhancement of Raman signal. This is in accordance with previously reported researches [2, 38]. Raman signal of *S. aureus* slightly reduced at higher concentration of Au-FePt@SiO_2_-N (190.4–238 μM) which is similar with the result of detecting adenine in Fig. 6.

**Fig. 8:**
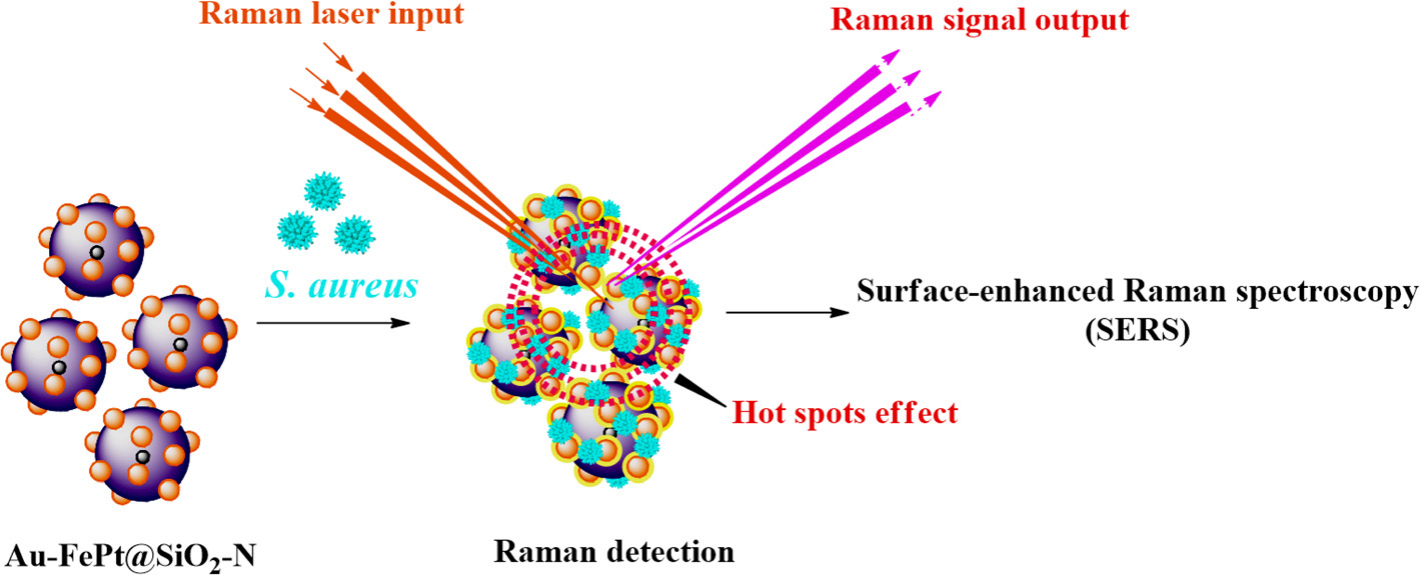
Schematic mechanism of magnetic separation and SERS detection of *S. aureus* using Au-FePt@SiO_2_-N SERS platform

## Conclusions

We fabricated multifunctional core-shell nanostructures of gold nanoparticles-decorated silica encapsulated FePt (Au-FePt@SiO_2_-N), which could be developed in the SERS detection, such as small biomolecules (adenine) and pathogenic bacteria (*S. aureus*). SERS spectra of adenine and *S. aureus* obtained from the Au-FePt@SiO_2_-N core-shell nanoparticles showed finger-print peaks with significant enhancement on the Raman signal. Furthermore, the magnetic core of FePt nanoparticles could be applied in the rapid magnetic separation of the biomolecules and bacteria and achieve the condensed effect. Therefore, the novel SERS platform of core-shell structure of Au-FePt@SiO_2_ nanoparticles (Fig. 8) would provide the potential to apply in the magnetic separation and bio-sensing at the same time, which could save the diagnosis time significantly.

## Additional file

Additional file 1:
**TEM images of core-shell nanoparticles.** **Figure S1.** TEM images of (A) FePt@SiO_2_-N and (B) gold nanoparticles (scale bar: 50 nm). **Figure S2.** TEM images of Au-FePt@SiO_2_-N with various EDS concentration: (A) 0 mM, (B) 0.1 M, (C) 0.2 M, (D) 0.3 M, (E) 0.4 M and (F) 0.5 M (scale bar: 50 nm). **Figure S3.** TEM images of Au-FePt@SiO_2_-N (0.3 M) with various gold concentration: (A) 0 μM, (B) 47.6 μM, (C) 95.2 μM, (D)142.8 μM, (E)190.4 μM, and (F) 238 μM (scale bar, 100 nm)
