# Supplementary material for: Core-shell of FePt@SiO2-Au magnetic nanoparticles for rapid SERS detection
Source: Nanoscale Res Lett. 2015 Oct 22;10:412. doi: 10.1186/s11671-015-1111-0 (PMC4614849; doi:10.1186/s11671-015-1111-0)
Supplement: Additional file 1: — TEM images of core-shell nanoparticles. Figure S1. TEM images of (A) FePt@SiO2-N and (B) gold nanoparticles (scale bar: 50 nm). Figure S2. TEM images of Au-FePt@SiO2-N with various EDS concentration: (A) 0 mM, (B) 0.1 M, (C) 0.2 M, (D) 0.3 M, (E) 0.4 M and (F) 0.5 M (scale bar: 50 nm). Figure S3. TEM images of Au-FePt@SiO2-N (0.3 M) with various gold concentration: (A) 0 μM, (B) 47.6 μM, (C) 95.2 μM, (D)142.8 μM, (E)190.4 μM, and (F) 238 μM (scale bar, 100 nm) [file 11671_2015_1111_MOESM1_ESM.docx]

**Supplementary Information**

**Figure S1** TEM images of (A) FePt@SiO_2_-N and (B) gold nanoparticles (scale bar: 50 nm)

**Figure S2** TEM images of Au-FePt@SiO_2_-N with various EDS concentration: (A) 0 mM, (B) 0.1 M, (C) 0.2 M, (D) 0.3 M, (E) 0.4 M and (F) 0.5 M (scale bar: 50 nm)

**Figure S3** TEM images of Au-FePt@SiO_2_-N (0.3M) with various gold concentration: (A) 0 μM, (B) 47.6 μM, (C) 95.2 μM, (D)142.8 μM, (E)190.4 μM and (F) 238 μM (scale bar: 100 nm)
